# Phage Display for the Generation of Antibodies for Proteome Research, Diagnostics and Therapy

**DOI:** 10.3390/molecules16010412

**Published:** 2011-01-10

**Authors:** Thomas Schirrmann, Torsten Meyer, Mark Schütte, André Frenzel, Michael Hust

**Affiliations:** Technische Universität Braunschweig, Institute of Biochemistry and Biotechnology, Department of Biotechnology, Spielmannstr. 7, 38106 Braunschweig, Germany

**Keywords:** antibody, scFv, phage display, cancer, diagnostics, proteome research, panning

## Abstract

Twenty years after its development, antibody phage display using filamentous bacteriophage represents the most successful *in vitro* antibody selection technology. Initially, its development was encouraged by the unique possibility of directly generating recombinant human antibodies for therapy. Today, antibody phage display has been developed as a robust technology offering great potential for automation. Generation of monospecific binders provides a valuable tool for proteome research, leading to highly enhanced throughput and reduced costs. This review presents the phage display technology, application areas of antibodies in research, diagnostics and therapy and the use of antibody phage display for these applications.

## 1. Polyclonal and Monoclonal Antibodies

Production of polyclonal antibodies by immunisation of animals is a method established for more than a century. The first antibody serum was produced in horses and directed against diphtheria [1]. Hybridoma technology was the next milestone, allowing the production of monoclonal antibodies by fusion of an immortal myeloma cell with an antibody-producing spleen cell [2]. However, hybridoma technology has some limitations: possible instability of the aneuploid cell lines, most of all its limited possible application to generate human antibodies and its inability to provide antibodies against toxic or highly conserved antigens [3]. Murine antibodies are limited regarding a therapeutic application, because repeated administration can cause human anti-mouse antibody reaction (HAMA) [4,5]. The immunogenicity can be reduced by chimerisation or humanisation [6,7,8,9,10]. There are two possibilities for the generation of human antibodies using hybridoma technology: the first is the generation of human hybridomas. This experimentally very difficult technology depends on the availability of B-lymphocytes from e.g., infected or immunised humans. Therefore, this technology is also restricted due to ethical reasons. The alternative is antibody generation using transgenic mice or cows which comprise human immunoglobulin loci instead of murine Ig loci. After immunisation, these transgenic animals produce human antibodies [11,12,13,14,15]. This technology can generate antibodies for a limited amount of therapeutic targets, but in our opinion the immunisation of animals does not allow generation of antibodies for proteome projects, because of the effort required to immunise and handle thousands of animals.

## 2. Antibody Phage Display

An alternative for the generation of human antibodies is antibody phage display, which is completely independent from any immune system and uses an *in vitro* selection process. The first antibody gene repertoires in phage were generated and screened by using the lytic phage Lambda [16,17], however with limited success. The display method most commonly used today is based on the ground breaking work of Georg P. Smith [18] on filamentous phage display. Here, the genotype and phenotype of oligo-peptides were linked by fusing the corresponding gene fragments to the minor coat protein III gene of the filamentous bacteriophage M13. The resulting peptide::pIII fusion protein is displayed on the surface of phage allowing affinity purification of the peptide and its corresponding gene. In the same way, antibody fragments fused to pIII can be presented on the surface of M13 phage [19,20,21,22,23].

Due to limitations of the *E. coli* folding machinery, only antibody fragments like single chain fragment variable (scFv), fragment antigen binding (Fabs), the variable heavy domain of camels (VHH) or humans (dAbs), which bind specifically without a corresponding light chain variable domain, are used routinely for antibody phage display [24,25,26]. Production of immunoglobulin G (IgG) in *E. coli* is only possible in rare cases [27,28]. In our opinion, the IgG phage display cannot compete with Fab or scFv display and the additional Fc part is not a benefit for the display of binders. 

For the expression of antibody::pIII fusion proteins for phage display, two different genetic systems have been developed. First, antibody genes have been directly inserted into the phage genome fused to the wildtype M13 phage protein III (pIII) gene [20]. However, most of the successful systems uncouple antibody expression from phage propagation by providing the genes encoding the antibody::pIII fusion proteins on a separate plasmid (called “phagemid”). This phagemid contains a phage morphogenetic signal for packaging the vector into the assembled phage particles. Hence, the antibody gene replication and expression is uncoupled from the phage replication cycle, leading to a higher genetic stability and a simplification of the antibody gene library amplification. Using the phagemid system, a helper phage is needed for the production of antibody phage particles [19,21,22,23].

## 3. Selection of Antibodies

**Figure 1:**
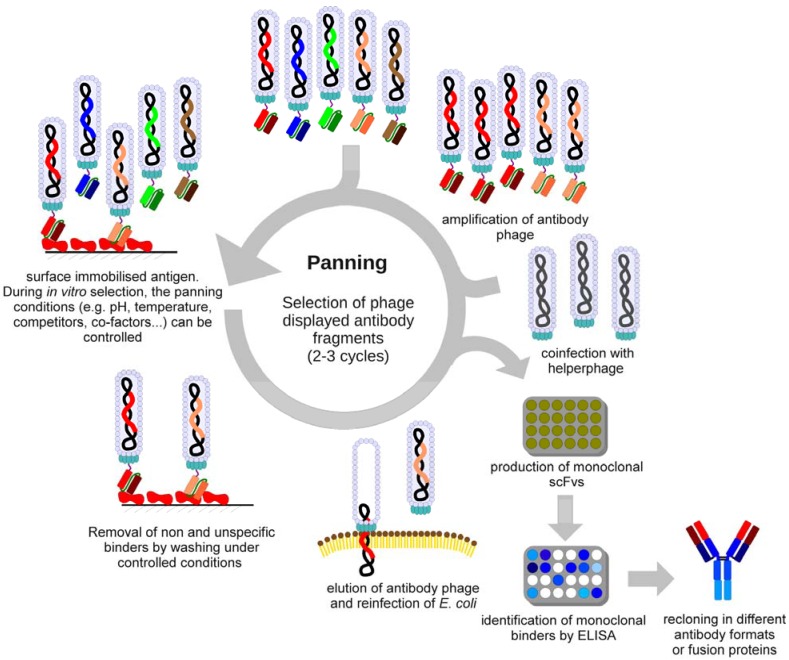
Schematic overview of the selection of antibodies (“panning“) by phage display.

*In vitro* isolation of antibody fragments from antibody gene libraries by their binding activity is called “panning”, referring to the gold washer‘s tool [29] (Figure 1). 

The antigen is immobilised on a solid surface such as nitrocellulose [30], magnetic beads [31], column matrices [19], plastic surfaces like polystyrene tubes [32] or 96 well microtitre plates [23] and incubated with antibody phage of the antibody gene library. During this incubation step, physical (e.g., temperature), chemical (e.g., pH) and biological (e.g., competitor) parameters can be controlled to select antibodies able to bind the antigen under these defined conditions. Antibody phage particles which bind weakly to the antigen and the vast excess of non-binding antibody phage are removed by stringent washing. Specifically binding antibody phage are eluted (e.g., by trypsin or pH shift) and reamplified by infection of *E. coli*. Subsequently, the phagemid bearing *E. coli* are infected with a helperphage to produce new antibody phage which can be used for further panning rounds until a significant enrichment of antigen specific phage is achieved. Usually two or three panning rounds, and sometimes up to six, are necessary to select specifically binding antibody fragments. After panning, soluble monoclonal antibody fragments or antibody phage are produced and specific antigen binding is analysed by ELISA to identify individual binders. Afterwards, these individual binders can be sequenced and further biochemically characterised [3,33,34,35,36]. This panning process can also be performed in a high-throughput manner [36,37,38]. Because the gene sequence of the binder is available, the antibody – depending on the desired application – can be reconverted into different antibody formats (e.g., scFv-Fc fusion or IgG) and produced in different production hosts [39,40]. Affinity, but also the stability, of the antibodies selected by phage display can be increased by additional *in vitro* affinity maturation steps [41,42,43].

## 4. Antibody Gene Libraries

For scientific or medical applications, different types of antibody gene libraries can be constructed. Immune libraries are constructed from immunised donors and typically generated and used in medical research to get an antibody against one particular target antigen, e.g., of an infectious pathogen [21], [44,45,46]. V-genes of these libraries contain hypermutations and are affinity matured. Naive, semi-synthetic and synthetic libraries have been subsumed as “single-pot” libraries, as they are designed to isolate antibody fragments binding to every possible antigen, at least in theory [47]. Naive libraries are constructed from rearranged V genes from B cells (IgM) of non-immunized donors. Examples for this library type are the naive human Fab library constructed by de Haard *et al.* [48] and the HAL scFv libraries [40]. Semi-synthetic libraries are constructed from unrearranged V genes from pre B cells (germline cells) [49] or from one antibody framework [50] in which one or several complementary determining regions (CDR), but always the CDR H3, are randomised. A combination of naive and synthetic repertoire was used for the FAB310 antibody gene library. In this library, light chains from autoimmune patients were combined with a Fd fragment containing synthetic CDR1 and CDR2 in the human VH3-23 framework and naive CDR3 regions, originated from autoimmune patients [25]. Fully synthetic libraries are made of human frameworks with randomised CDR cassettes [51,52]. To date, “single-pot” antibody libraries with a theoretical diversity of up to 10^11^ independent clones have been generated [53] to serve as a molecular repertoire for phage display selection procedures.

## 5. Antibodies for Basic Research

Antibodies are essential tools for biological basic research. Many methods require antibodies to specifically detect proteins or other molecules. Often used standard methods are immunoblot [54], [55], ELISA [56], immunofluorescence microscopy [57], flow cytometry [58], purification of molecules or cells by affinity chromatography [59], or more recent microarrays [60,61]. The first antibodies derived from phage display for basic research have reached the market (e.g., anti-CD256 from Axxora).

An important field for antibodies in basic research is proteome research. After sequencing of the human genome, including the genome of individual persons, the research focus shifted to the analysis of gene products. The human genome encodes about 20,000-25,000 protein encoding genes [62,63,64]. Due to alternative mRNA splicing and post-translational modifications, e.g., glycosylation, phosphorylation *etc*., the number of different human proteins is supposed to exceed the amount of genes severalfold [65]. By different interpretation of the same genetic content, dramatic morphological variations are achieved, e.g., between caterpillar and butterfly. Therefore, for each gene tools are necessary to investigate amount, localisation and function of its gene products, as the genotype defines the species of an organism, but the proteome defines the phenotype. Here, antibodies are a key tool for the decryption of the human proteome [47,66,67,68,69]. For this purpose, pilot projects to generate antibodies to the complete human proteome were performed and complete antibody generation pipelines were developed [40,70,71]. The information about individual binders against human proteins are stored and shared in the “Antibodypedia” project (www.antibodypedia.org) [72] while information about analysed human proteins are given in the “Human Proteome Atlas” (www.proteinatlas.org) [73].

In our opinion, only *in vitro* technologies like phage display or ribosome display [74] can fulfill the requirement to generate binders like antibody fragments or alternative binders e.g., DARPins [75] or anticalins [76] to the complete human proteome. In comparision to the immunisation of animals, *in vitro* technologies are automatable [36,37,38]. Furthermore, *in vitro* technologies can deliver an unlimited and defined resource of binders. Here, the storage and the provision of the binders can be achieved by storage of the corresponding DNA sequence which is directly available.

## 6. Antibodies for Diagnostics

There are three general ways to use antibodies for diagnostics. The first way is the detection of antigens using antibodies [77] and the second, but reversed way is the detection of serum antibodies using antigens [78]. The third way is the competition assay, here, e.g., antigens are detected by serum antibodies which compete with a defined antibody preparation [79]. The advantage of an antibody competition assay is its species independence [80]. Other prominent technologies used for diagnostics are lateral flow strip assays [81,82], bead based assays such as Luminex [83], flow cytometry [58,84], the hemagglutination assay [85], and more recently, the proximity ligation assay [86] or molecular imaging [87]. An interesting diagnostic method is the direct use of phage particles fused to antibodies [88] for diagnostics. Sensitivity of the diagnostic assay can be enhanced by detection of the about 2700 copies of pVIII of an antibody phage by secondary antibodies [89,90]. Antibody phage particles can also be used for immuno-PCR [91].

To date, there are many examples for the application of antibodies derived by phage display for diagnostics [92,93,94]. A big advantage of antibody phage display for diagnostics is the direct access to the genetic information of the binder, allowing a fast adaption of the antibody format e.g., IgG, scFv-Fc, biotinylated antibody or scFv-phoA fusion [40] to the desired diagnostic assay.

Phage display allows also the identification of immunogenic proteins, e.g., from pathogens [95,96,97]. Combining antibody phage display with antigen phage display all steps from identification of antigens to the generation of antibodies against these antigens for diagnostics are available.

## 7. Antibodies for Therapeutical Applications

In 2010, 28 therapeutic monoclonal antibodies were approved by the FDA and for further approvals are pending (US Food and Drug Administration) (http://www.landesbioscience.com/journals/ mabs/about). Most of the accepted therapeutic antibodies are for cancer and autoimmune diseases [98]. Mechanisms of therapeutic antibodies are manifold and include neutralisation of substances, e.g. toxins [44] or cytokines like tumor necrosis factor (TNF) alpha [99], blocking of receptors like epidermal growth factor receptor (EGFR) [100], binding to cells and modulating the host immune system [101], or combinations of these effects [102].

In 1986, the first approved antibody muronomab-CD3 (tradename Orthoclone OKT3^®^) was directed against the T-cell marker CDR to minimise possible organ rejection after organ transplantations. This antibody was still of murine origin [101]. The next therapeutic antibodies were chimeric. Here, constant domains of an antibody are human and the variable domains are murine. Examples are infliximab (tradename Remicade^®^) against TNF-alpha for treatment of rheumatoid arthritis and other autoimmune diseases [103] or the anti-EGFR antibody cetuximab (tradename Erbitux^®^) for colorectal cancer treatment [104]. Later, antibodies were humanised. Here, also the murine framework regions of the variable domains were replaced by their human counterpart. Examples are trastuzumab (tradename Herceptin^®^) an anti-human epidermal growth factor receptor 2 (HER2) binder for treatment of breast cancer [105]. In 2002, the first fully human antibody was commercially available. The anti-TNF-alpha antibody adalimumab (tradename Humira^®^) was isolated using antibody phage display by guided selection using a murine antibody as template [10,99]. Other FDA-approved human antibodies are the anti-EGFR antibody panitumumab (tradename Vectibix^®^) [106] and golimumab (tradename Simponi^®^) [107] directed against TNF-alpha. Further examples are canakinumab (tradename Ilaris^®^), an interleukin-1β (Il-1β)-specific antibody for treatment of cryopyrin-associated periodic syndromes [108], and the anti-IL12/23 antibody ustekinumab (tradename Stelara^®^) for treatment of psoriasis [109]. These human antibodies are derived from transgenic mice expressing the human antibody gene repertoire.

To date, several therapeutic antibodies derived from phage display are in clinical development [33,98,110,111]. Most phage display derived antibodes are generated by CAT (now MedImmune, part of AstraZeneca), Morphosys and Dyax. An example for the generation of human antibodies by phage display and further development is belimumab (tradename Benlysta^®^). This antibody was generated by CAT for treatment of autoimmune diseases, mainly systemic lupus erythematosus (SLE). This antibody was selected from the Vaughan library [112] against the B lymphocyte stimulator (BLyS). Afterwards, the initial binders were ranked by the efficiency to block BLyS binding to the BLyS receptor in cell based assays. The best scFvs were converted into the IgG format and neutralisation efficiency of the IgG were analysed again [113]. This step is important, because the affinity of some antibodies is decreased after converting from the scFv to the IgG format [113,114]. Two lead scFvs were further optimised by randomisation of the CDR3 of the heavy chain and selection on BLyS by phage display. ScFv with an increased affinity were again converted into the IgG format and analysed regarding neutralisation efficiency. One clone "LymphoStat-B" was further analysed in biochemical, *in vitro*, *in vivo* assays using mice and cynomolgus monkeys and in clinical studies [113,115]. The anticipated approval date for Benlysta by the US Food and Drug Administration is 2011 (www.fda.gov). A further phage display derived antibody which is currently under review by the FDA is raxibacumab. This antibody is binding to the protective antigen (PA83) of *Bacillus anthracis* and was also developed by CAT [116].

The advantage of antibody phage display is the direct isolation of human antibodies in comparison to antibodies generated by mouse hybridoma technology where a laborious humanisation of the lead candidates is necessary. When using transgenic mice comprising the human antibody repertoire human antibodies are also isolated, but antibody generation is limited by the immune system, whereas antibody phage display circumvents this limitation. On the other hand, the advantage of immunisation is the affinity maturation of the desired antibodies resulting in antibodies with a higher affinity compared to antibodies selected from naive antibody gene libraries. But phage display selected antibodies can be easily further stability and affinity optimised. In our opinion, the straightforward method of choice is to introduce random mutations in the antibody gene by error prone PCR, followed by the construction of an antibody gene mutation phage display library. Binders with the highest affinity and stability will be selected by off-rate panning [41,114]. Independent of all antibody selection strategies, the rise and fall of a therapeutic antibody depends on the target, e.g. cancer marker protein [114]. Antibody phage display is generating and will generate the leads for many antibody based therapeutics in the next years.
